# Inhibitory effects of superoxide dismutase 3 on IgE production in B cells

**DOI:** 10.1016/j.bbrep.2022.101226

**Published:** 2022-02-03

**Authors:** Gaurav Agrahari, Shyam Kishor Sah, Min Jung Lee, Chul Hwan Bang, Yeong Ho Kim, Hey-Young Kim, Tae-Yoon Kim

**Affiliations:** a Laboratory of Dermato-Immunology, College of Medicine, The Catholic University of Korea, 06591, Seoul, Republic of Korea; b Department of Reconstructive Sciences, Center for Regenerative Medicine and Skeletal Development, UConn Health, USA

**Keywords:** Superoxide dismutase 3, Immunoglobulin E, B cells, Class switch recombination, Signaling pathways

## Abstract

Immunoglobulin E (IgE) functions as a first-line defense against parasitic infections. However, aberrant production of IgE is known to be associated with various life-threatening allergic diseases. Superoxide dismutase 3 (SOD3) has been found to suppress IgE in various allergic diseases such as allergic conjunctivitis, ovalbumin-induced allergic asthma, and dust mite-induced atopic dermatitis-like skin inflammation. However, the role of SOD3 in the regulation of IgE production in B cells remains elusive. In this study, we investigated the effect of SOD3 on LPS/IL-4 and anti-CD40/IL-4-mediated secretion of IgE in murine B cells. Our data showed that SOD3 can suppress both LPS/IL-4 and antiCD40/IL-7-induced IgE secretion in B cells isolated from both wild-type (*SOD3*^*+/+*^) and SOD3 knock-out (*SOD3*^*−/−*^) mice. Interestingly, B cells isolated from *SOD3*^*−/−*^ mice showed higher secretion of IgE, whereas, the use of DETCA, a known inhibitor of SOD3 activity, reversed the inhibitory effect of SOD3 on IgE production. Similarly, SOD3 was found to reduce the proliferation, IgE isotype switch, ROS level, and CCL17 and CCL22 productions in B cells. Furthermore, SOD3 was found to suppress both LPS/IL-4 and anti-CD40/IL-4-mediated activation of downstream signaling such as JAK1/JAK3, STAT6, NF-κB, p38, and JNK in B cells. Taken together, our data showed that SOD3 can be used as an alternative therapy to restrict IgE-mediated allergic diseases.

## Highlights

• SOD3 suppresses LPS/IL-4 and anti-CD40/IL-4-induced secretion of IgE in B cells
• SOD3 reduces the expression of IgE isotype class switch recombination genes.
• SOD3 suppresses the LPS/IL-4 and anti-CD40/IL-4-induced superoxide production.
• SOD3 suppresses the LPS/IL-4 and anti-CD40/IL-4-induced chemokines secretions.
• SOD3 modulate JAK-STAT, p38, JNK, and NF-κB signaling pathways in B cells.

## 1 Introduction

Immunoglobulin E (IgE) is the immunoglobulin type that acts as a first-line defense against parasitic infections. However, inappropriate regulation of IgE is found to be associated with various allergic diseases and can lead to severe life-threatening anaphylaxis [1]. Serum IgE levels are found to be elevated in various allergic diseases such as asthma, allergic rhinitis, atopic dermatitis, contact dermatitis, and urticaria [2]. The production of IgE is tightly regulated by class switch recombination (CSR) and requires major two signals: T helper cell-B cell interaction through CD40L/CD40 accompanied by IL-4 secreted by activated T cells. The interaction of CD40L and CD40 leads to the activation of nuclear factor κB (NFκB), p38 mitogen-activated protein kinase (p38), and c-jun-NH2-kinase (JNK) whereas binding of IL-4 to its receptor complexes activates downstream Janus kinases (JAK1/JAK3) and the following signal transducer and activator of transcription 6 (STAT6) phosphorylation [[3], [4], [5]]. These signals work synergistically for the transcription and synthesis of IgE [6]. Therefore, NF-κB, p38, JNK, and JAK-STAT signaling pathways can be targeted to alleviate the inappropriate production of IgE and hyper IgE-mediated allergic syndrome.

Superoxide dismutase 3 (SOD3), also known as extracellular superoxide dismutase is responsible for the maintenance of redox homeostasis in the extracellular matrix of various tissues [7]. Along with antioxidative properties, the anti-inflammatory function of SOD3-mediated through both enzymatic and non-enzymatic manner have been well established in various disease models [[8], [9], [10], [11]]. In addition, some studies also revealed the anti-allergic function of SOD3 in various allergic conditions. In experimental allergic conjunctivitis and ovalbumin-induced allergic asthma murine models, treatment with SOD3 showed reduced serum level of OVA-induced IgE [12,13]. Similarly, the treatment with SOD3 also found to suppress the serum IgE levels in house dust mite-induced atopic dermatitis-like skin inflammation [14]. Thus, SOD3 was found to regulate the production of IgE during various allergic conditions. However, the direct role of SOD3 in regulating the production of IgE is still unclear.

In this study, we aimed to determine the effect of SOD3 on IgE production in B cells. Primary naïve B cells were differentiated for IgE production and the effects of SOD3 on IgE production and underlying effects on signaling were investigated. Our data showed that SOD3 limits the IgE production in B cells with reduced proliferation and IgE isotype class switching. Thus, our study provides supportive information regarding the use of SOD3 in hyper-IgE-mediated allergic conditions.

## 2 Materials and methods

### 2.1 B cell isolation and treatment

All procedure of animal research was performed in accordance with the Laboratory Animals Welfare Act, the Guide for the Care and Use of Laboratory Animals, and the Guidelines and Policies for Rodent experiment provided by the IACUC (Institutional Animal Care and Use Committee) in the school of medicine, The Catholic University of Korea (Approval number: CUMS-2019-0285-03). Following the approval from The Catholic Ethics Committee of Catholic University of Korea, primary B cells were isolated from mouse spleen (male, 8–12 weeks old) of C57/BL6 wild-type (*SOD3*^*+/+*^) and whole-body SOD3 knock-out (*SOD3*^*−/−*^) mice using the MACS B cell isolation kit (130-090-862, Miltenyi Biotec, Teterow, Germany) following manufacturer instructions. Purified B cells were then rested at least 2 h on ice and then 2 × 10^5^ B cells were cultured in flat-bottom 96-well plates. The cells were cultured in Roswell Park Memorial Institute (RPMI)-1640 medium (CA059-050; Gendepot, Houston, Tx) supplemented with 10% fetal bovine serum, 100 U/mL penicillin, and 100 μg/mL streptomycin (CA 005–010; Gendepot) at 37 °C in a humidified incubator.

For experimental setup, isolated B cells were pre-treated with recombinant human SOD3 (rh SOD3; 100U/mL, 200U/mL, and 300U/mL) for 1 h. The cells were then treated with a combination of either 10 μg/mL of LPS (L6529, Sigma-Alrich) and 10 ng/mL of rh mouse IL-4 (MIL 4–25, JW CreaGene Inc.) or 5 μg/mL of anti-CD40 (Cyt-472, Prospec) and 10 ng/mL of rh mouse IL-4 (MIL 4–25, JW CreaGene Inc.) for 5 days.

### 2.2 ELISA of IgE production

The levels of IgE release were measured from cell-free supernatant by ELISA method using IgE mouse ELISA kit (ab157718, Abcam) according to the manufacturer's instructions. Briefly, 100 μl of each standard and samples were added into pre-coated wells and were incubated at room temperature for 30 min. After incubation, the contents of the well were aspirated and washed with the provided 1X wash buffer for 5 times. Following washing, 100 μl of 1X enzyme-antibody conjugated were added to each well and the plates were incubated in dark for 30 min. The plates were then washed and 100 μl of TMB substrate solution were added to each well and incubated in dark at room temperature for 10 min. After 10 min, 100 μl of stop solution were added into each well, and absorbance was taken at 450 nm.

### 2.3 Cell proliferation assay

Isolated naïve B cells were plated in 96-well plates at 2 × 10^5^ cells/mL in 100 μL per well of culture media. Cells were then pre-treated with SOD3 for 1 h followed by treatment with either LPS/IL-4 or anti-CD40/IL-4 for 5 days. Cell proliferation was measured with BrdU cell proliferation assay kit (Catalog No. 2750, Millipore) following the manufacturer's instruction.

### 2.4 ELISpot assay

The number of IgE secreting cells was determined by using Mouse IgE ELISpotBASIC kit (3815-2A, MABTECH AB, USA) following the manufacturer's instructions. Briefly, provided PVDF plates were activated with 50 μl of 70% ethanol per well for 2 min. The plates were washed 5 times with sterile water and incubated overnight with 100 μl of anti-IgE antibody at 4 °C. The next day, coated antibodies were aspirated and washed with sterile water. The wells were then blocked with 200 μl of culture medium and incubated at room temperature for 30 min. After blocking, cell suspension with indicated stimuli was added to each well and incubated for 5 days at 37 °C in a humidified chamber with 5% CO_2_. After 5 days of incubation, the cells were removed and the wells were washed 5 times with 200 μl of PBS/well. Detection antibodies were added to each well and incubated at room temperature for 2 h. The wells were then incubated with 100 μl of streptavidin-ALP for 1 h at room temperature. Substrate solution was added and developed distinct spots were analyzed with the microscope.

### 2.5 ROS measurement

Isolated B cells were first pre-treated with SOD3 (100 U, 200 U, and 300 U/mL) for 1 h. The cells were then treated with either LPS/IL-4 or anti-CD40/IL-4 and 5 μM of dihydroethidium (DHE) (D1168, Invitrogen), and incubated in dark at 37 °C for 10, 30, and 60 min. Fluorescents were measured at an excitation of 500 nm and emission of 600 nm by fluorimeter.

### 2.6 Western blot analysis

Cells were harvested after 5 days of incubation at 37 °C in humidified incubator and were lysed in ice-cold radioimmunoprecipitation (RIPA) lysis buffer (Catalog No. 89901, Thermo Scientific, Rockford, USA) containing protease and phosphatase inhibitor cocktails (Roche Diagnostic, Germany). Protein concentrations were determined by BCA protein assay Kit (Catalog No. 23225, Thermo Scientific, Rockford, USA) as described by manufacturer instructions. Equal amounts of proteins were separated by sodium dodecyl sulfate-polyacrylamide gel electrophoresis (SDS-PAGE) and transferred to polyvinylidene difluoride membrane (PVDF). After blocking, membranes were incubated with primary antibodies such as p-p38 (9211S, Cell Signaling), p38 (9212S, Cell Signaling), P-JNK (9251S, Cell Signaling), JNK (9252S, Cell Signaling), P-JAK1 (3331S, Cell Signaling), JAK1 (3332S, Cell Signaling), P-JAK3 (5031S, Cell Signaling), JAK3 (8863S, Cell Signaling) P-STAT6 (9361S, Cell Signaling), STAT6 (9362S, Cell Signaling), p-NF-κBp65 (SC-136548, Santa Cruz), NF-κBp65 (SC-109, Santa Cruz) and GAPDH (SC-32233, Santa Cruz) at 1:1000 dilutions for overnight at 4 °C. Membranes were then washed and incubated with horseradish-peroxidase conjugated secondary antibodies (1:5000 dilutions) for 2 h at room temperature. The blots were then detected with western blot detection kit (WesternBrightTMECL, USA).

### 2.7 cDNA synthesis and reverse-transcriptase PCR (RT-PCR) analysis

Total RNA was isolated from cells by using TRIzol reagent (Catalog No. 15596018, Life Technologies, Invitrogen). Complementary DNA was synthesized from 1 μg of total RNA using the PrimeScript™ RT reagent Kit (RR047A, Clontech Takara Bio INC, Japan) and RT-PCR was performed using LightCycler 96 (Roche Diagnostics, Mannheim, Germany). Glyceraldehyde 3-phosphate dehydrogenase (GAPDH) was used as an endogenous control. The amplified products were analyzed by electrophoresis in a 2% agarose gel. The used primers and their sequences can be found in supplementary sections (Table S1).

### 2.8 SOD3 purification and activity assay

SOD3 was purified as previously described [15]. Briefly, SOD3 plasmids were transfected into human embryonic kidney cells (HEK293E) and media were collected after every 48 h. The collected media were filtered and loaded on HiTrap Chelating HP column (GE Healthcare). The recombinant SOD3 was verified by western blot with SOD3 antibody as previously described [16]. SOD3 activity in the culture medium was analyzed by SOD assay kit-WST as per the manufacturer's instruction (S311, Dojindo Molecular Technologies, Japan).

### 2.9 Statistical analysis

Statistical differences were analyzed by one-way analysis of variance (ANOVA) followed by Tukey test. All results represent three independent experiments. P < 0.05 was considered statistically significant (*p < 0.05, **p < 0.01, ***p < 0.001).

## 3 Results

### 3.1 Effect of SOD3 on IgE production in LPS/IL-4 and anti-CD40/IL-4-stimulated B cells

**Fig. 1:**
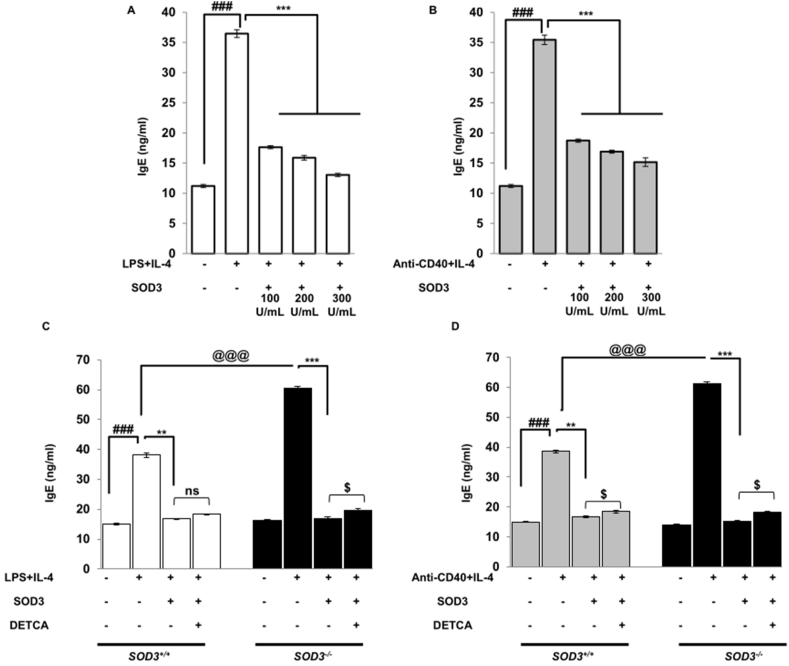
**SOD3 suppressed IgE secretion in B cells.** (A–B) B cells were pre-treated with SOD3 (100U, 200U and 300U/mL) for 1 h followed by treatment with either (A) LPS (10 μg/mL)/IL-4 10 ng/mL or (B) anti-CD40 (5 μg/mL)/IL-4 (10 ng/mL). The level of IgE secretion was determined after 5 days of incubation from cell-free supernatant by ELISA. (C–D) B cells were isolated from SOD3+/+ and SOD3^−/−^ mice and pre-treated with SOD3 (200U/mL). The cells were then treated with differentiating stimuli and DETCA (10 μM). The level of IgE was measured by ELISA. All experiments were performed in triplicates. Data are expressed as mean ± standard deviation. ^#^p < 0.05, ^##^p < 0.01, ^###^p < 0.001 (Control group vs. LPS/IL-4 or anti-CD40/IL-4-treated group); *p < 0.05, **p < 0.01, ***p < 0.001 (LPS/IL-4 or anti-CD40/IL-4-treated group vs. SOD3-treated group); ^@^p < 0.05, ^@@^p < 0.01, ^@@@^p < 0.001 (*SOD3*^*+/+*^ vs *SOD3*^*−/−*^).

Previously, SOD3 was found to suppress the serum IgE level during various allergic conditions [9,14,17]. Thus, we first aimed to determine the effect of SOD3 on IgE production *in vitro*. Here, we found that SOD3 significantly suppresses the production of IgE in B cells under both LPS/IL-4 and anti-CD40/IL-4 conditions (Fig. 1 A and B). To further determine SOD3-mediated inhibition of IgE production, we isolated the B cells from whole body SOD3 knock-out (*SOD3*^*−/−*^) mice and also used DETCA, a known inhibitor of SOD3 activity. Our data showed that the IgE production was relatively higher in B cells isolated from *SOD3*^*−/−*^ mice compared to wild-type (*SOD3*^*+/+*^) mice and the use of SOD3 downregulates the release of IgE in both *SOD3*^*−/−*^ and *SOD3*^*+/+*^ mice. In addition, DETCA rescued the inhibitory effect of SOD3 on IgE production in both *SOD3*^*+/+*^and *SOD3*^*−/−*^ mice under LPS/IL-4 and anti-CD40/IL-4 conditions (Fig. 1C and D).

### 3.2 Effect of SOD3 on proliferation in LPS/IL-4 and anti-CD40/IL-4-stimulated B cells

**Fig. 2:**
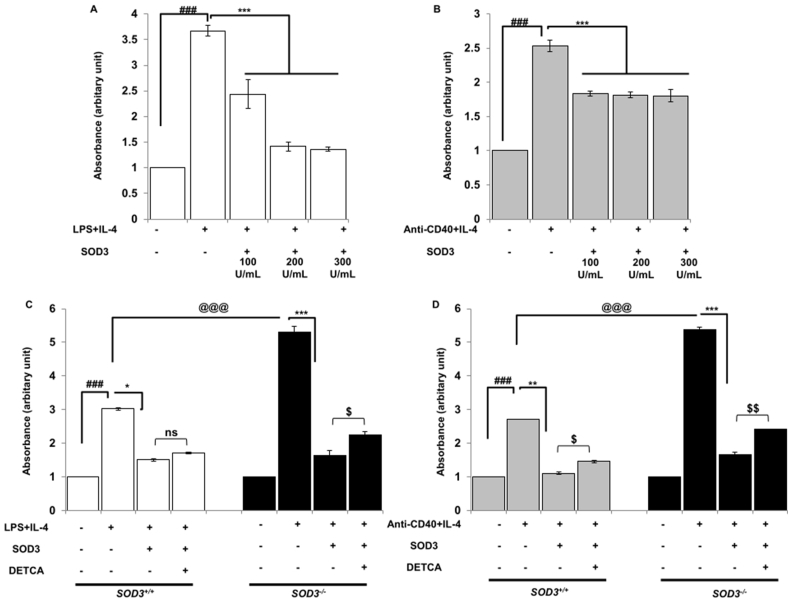
**Inhibitory effect of SOD3 on B cell proliferations.** (A–B) B cells were pre-treated with SOD3 (100U, 200U and 300U/mL) for 1 h followed by treatment with either (A) LPS (10 μg/mL)/IL-4 10 ng/mL or (B) anti-CD40 (5 μg/mL)/IL-4 (10 ng/mL). Cell proliferation was determined by BrdU assay. (C–D) B cells isolated from SOD3+/+ and SOD3^−/−^ mice were pre-treated with SOD3 (200U/mL) and then treated with differentiating stimuli and DETCA (10 μM). Proliferation was analyzed by BrdU assay. All experiments were performed in triplicates. Data are expressed as mean ± standard deviation. ^#^p < 0.05, ^##^p < 0.01, ^###^p < 0.001 (Control group vs. LPS/IL-4 or anti-CD40/IL-4-treated group); *p < 0.05, **p < 0.01, ***p < 0.001 (LPS/IL-4 or anti-CD40/IL-4-treated group vs. SOD3-treated group); ^@^p < 0.05, ^@@^p < 0.01, ^@@@^p < 0.001 (*SOD3*^*+/+*^vs *SOD3*^*−/−*^).

We next aimed to determine the effect of SOD3 on B cell proliferation as activated B cells are known to undergo proliferation. Here, we found that SOD3 significantly suppresses the B cell proliferation under both LPS/IL-4 and anti-CD40/IL-4 conditions (Fig. 2 A and B). In addition, B cells isolated from *SOD3*^*−/−*^ mice were highly proliferative as compared to *SOD3*^*+/+*^ mice. The use of SOD3 inhibited the proliferation of B cells isolated from *SOD3*^*−/−*^ and *SOD3*^*+/+*^ mice. Moreover, DETCA reversed the inhibitory effect of SOD3 on B cell proliferation (Fig. 2C and D).

### 3.3 Effect of SOD3 on IgE class switch recombination in LPS/IL-4 and anti-CD40/IL-4-stimulated B cells

**Fig. 3:**
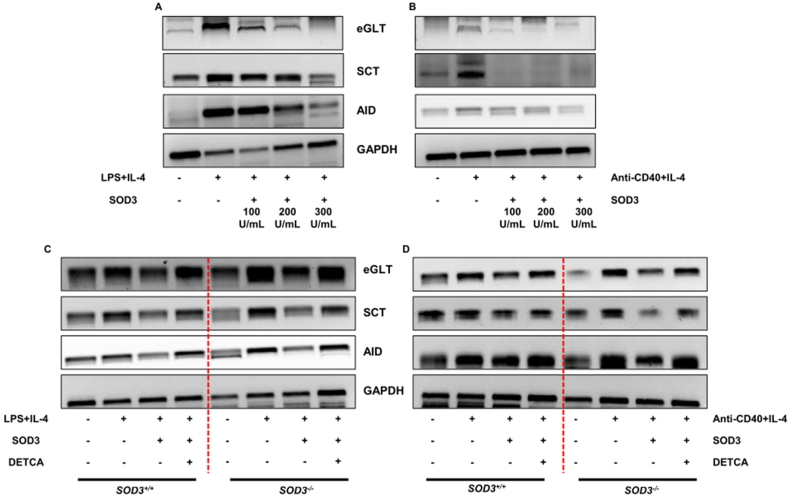
**SOD3 inhibits IgE isotype class switching in B cells.** (A–B) B cells were pre-treated with SOD3 (100U, 200U and 300U/mL) for 1 h followed by treatment with either (A) LPS (10 μg/mL)/IL-4 10 ng/mL or (B) anti-CD40 (5 μg/mL)/IL-4 (10 ng/mL). Genes involved in CSR were analyzed by RT-PCR. (C–D) B cells isolated from SOD3+/+ and SOD3^−/−^ mice were pre-treated with SOD3 (200U/mL) and then treated with differentiating stimuli and DETCA (10 μM). Genes involved in CSR were analyzed by RT-PCR. All experiments were performed in triplicates. Data are expressed as mean ± standard deviation. The band intensities of gel electrophoresis are presented in supplementary section (Fig. S1). Full length blots are presented in Fig. S2. ^#^p < 0.05, ^##^p < 0.01, ^###^p < 0.001 (Control group vs. LPS/IL-4 or anti-CD40/IL-4-treated group); *p < 0.05, **p < 0.01, ***p < 0.001 (LPS/IL-4 or anti-CD40/IL-4-treated group vs. SOD3-treated group); ^@^p < 0.05, ^@@^p < 0.01, ^@@@^p < 0.001 (*SOD3*^*+/+*^vs *SOD3*^*−/−*^).

B cells undergo class switch recombination (CSR) process for IgE production [3]. Thus, we next aimed to determine the effect of SOD3 on germline gene transcript (GLT), switch class transcript (SCT), and activation-induced cytidine deaminase (AID) at mRNA levels. Our data showed that SOD3 can significantly inhibit the B cells undergoing IgE isotype switching through the suppressed expression of GLT, SCT, and AID (Fig. 3 A and B). In addition, B cells isolated from *SOD3*^*−/−*^ mice showed higher expression of CSR genes than that of *SOD3*^*+/+*^ mice. Moreover, SOD3 suppressed the expressions of genes involved in CSR, and DETCA tried to nullify the effect of SOD3 in both *SOD3*^*−/−*^ and *SOD3*^*+/+*^ mice (Fig. 3C and D).

### 3.4 Effect of SOD3 on IgE secreting cells and ROS production in LPS/IL-4 and α-CD40/IL-4-stimulated B cells

**Fig. 4:**
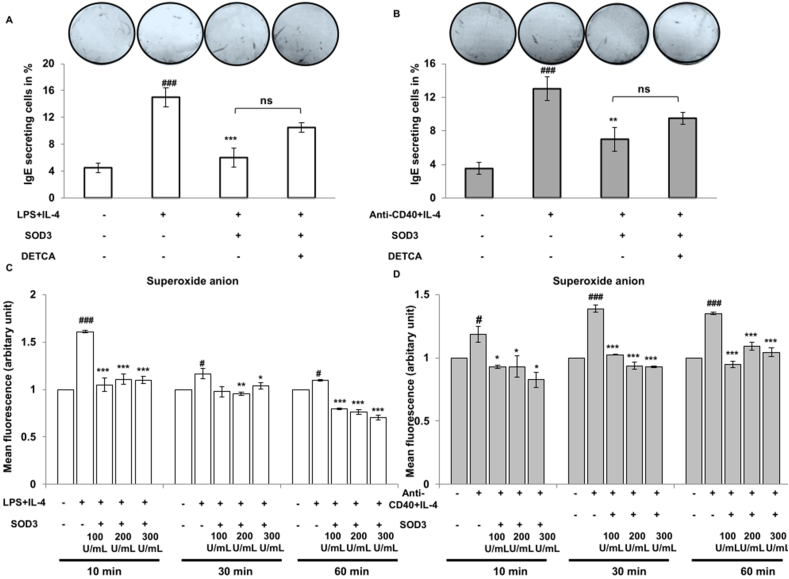
**SOD3 suppressed IgE secreting cells and ROS level.** (A–B) B cells were pre-treated with SOD3 (200U/mL) for 1 h followed by treatment with either (A) LPS (10 μg/mL)/IL-4 10 ng/mL and DETCA (10 μM) or (B) anti-CD40 (5 μg/mL)/IL-4 (10 ng/mL) and DETCA (10 μM). The numbers of IgE secreting cells were analyzed by ELISpot assay. (C–D) B cells were pre-treated with SOD3 (100U, 200U and 300U/mL) for 1 h followed by treatment with either (A) LPS (10 μg/mL)/IL-4 10 ng/mL or (B) anti-CD40 (5 μg/mL)/IL-4 (10 ng/mL) and DHE (5 μM) for 10, 30 and 60 min. ROS production were determined by fluorimeter at 500 nm; 600 nm ^#^p < 0.05, ^##^p < 0.01, ^###^p < 0.001 (Control group vs. LPS/IL-4 or anti-CD40/IL-4-treated group); *p < 0.05, **p < 0.01, ***p < 0.001 (LPS/IL-4 or anti-CD40/IL-4-treated group vs. SOD3-treated group).

Upon encountering with antigen and IL-4, activated B cells are known to differentiate into IgE secreting plasma cells [18]. Thus, we evaluated the effect of SOD3 on the number of IgE secreting cells by ELISpot assay. Here, we found that the number of IgE secreting cells was lower in SOD3-treated groups compared to both LPS/IL-4 and anti-CD4-/IL-4 groups, whereas the number of IgE secreting cells were comparatively higher in DETCA-treated group than that of SOD3-treated groups (Fig. 4 A and B). Engagement of B cell receptors with its ligand results in the production of ROS and can activate the downstream signaling for B cell activation and differentiation [19]. Thus, we next determined the effect of SOD3 on superoxide anion produced during B cell activation. Our data showed that SOD3 significantly downregulates LPS/IL-4 and anti-CD40/IL-4-induced superoxide anions in B cells (Fig. 4C and D).

### 3.5 Effect of SOD3 on chemokines production in B cells

**Fig. 5:**
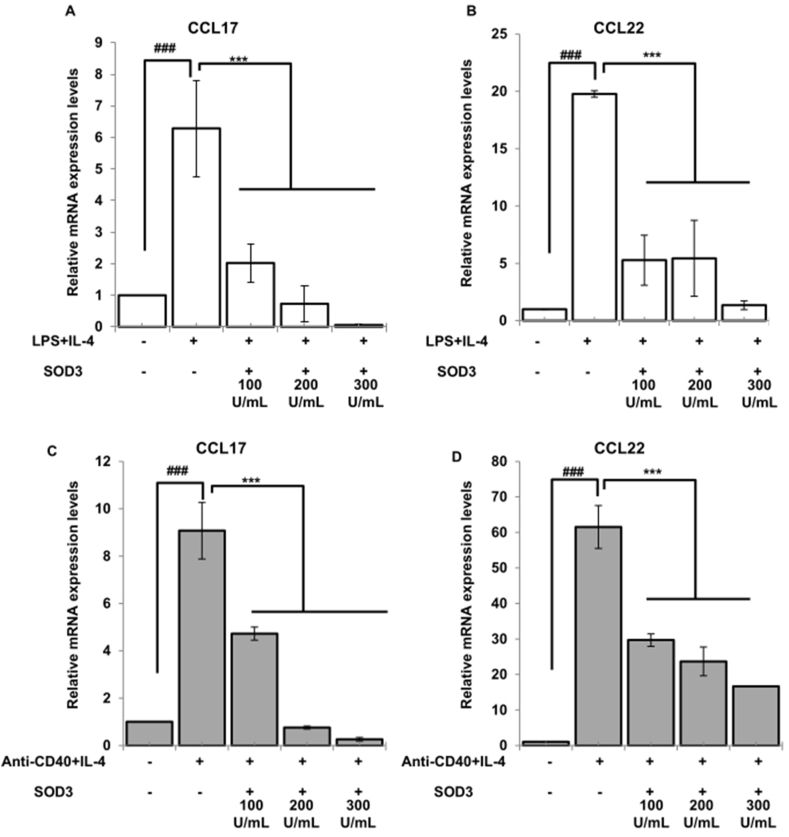
**SOD3 inhibits chemokines secretion in B cells.** (A–B) B cells were pre-treated with SOD3 (100U, 200U and 300U/mL) for 1 h followed by treatment with either (A) LPS (10 μg/mL)/IL-4 10 ng/mL or (B) anti-CD40 (5 μg/mL)/IL-4 (10 ng/mL). The levels of chemokines were analyzed by qRT-PCR. All experiments were performed in triplicates. Data are expressed as mean ± standard deviation. #p < 0.05, ##p < 0.01, ###p < 0.001 (Control group vs. LPS/IL-4 or anti-CD40/IL-4-treated group); *p < 0.05, **p < 0.01, ***p < 0.001 (LPS/IL-4 or anti-CD40/IL-4-treated group vs. SOD3-treated group).

Naïve B cells are also known to release chemokines such as CCL22 and CCL17 upon activation [[20], [21], [22]]. Thus, we next determined the effect of SOD3 on chemokines produced by activated B cells. Here, we found that SOD3 suppressed the expression levels of CCL17 and CCL22 chemokine in both LPS/IL-4 and anti-CD40/IL-4-stimulated B cells (Fig. 5A–D).

### 3.6 Effect of SOD3 on downstream signaling pathways

**Fig. 6:**
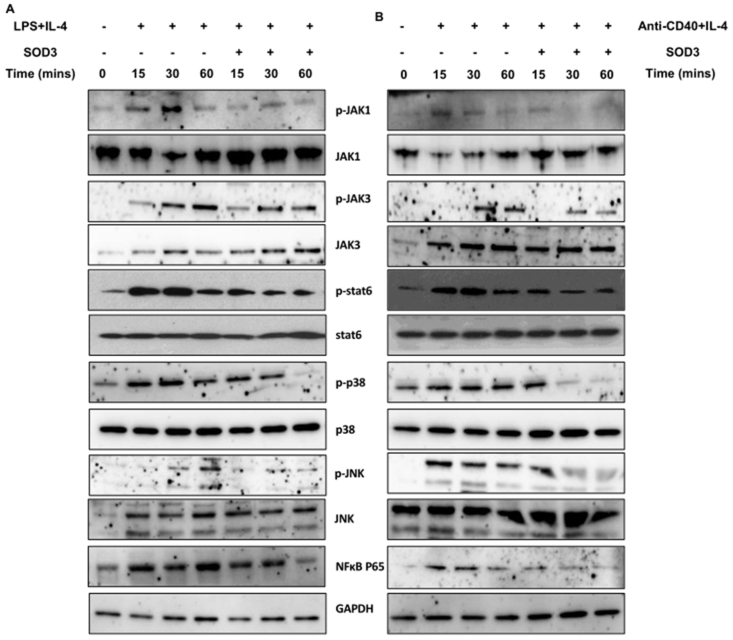
**SOD3 modulated downstream signaling pathways.** (A–B) B cells were pre-treated with SOD3 (200U/mL) for 1 h followed by treatment with either (A) LPS (10 μg/mL)/IL-4 10 ng/mL or (B) anti-CD40 (5 μg/mL)/IL-4 (10 ng/mL) for 15, 30 and 60 min. Activation of JAK1, JAK3, STAT6, p38, JNK and NF-κB were analyzed by western blot. All experiments were performed in triplicates. Data are expressed as mean ± standard deviation. The band intensities of western blots are presented in supplementary section (Fig. S3). Full length blots are presented in Figure S. #p < 0.05, ##p < 0.01, ###p < 0.001 (Control group vs. LPS/IL-4 or anti-CD40/IL-4-treated group); *p < 0.05, **p < 0.01, ***p < 0.001 (LPS/IL-4 or anti-CD40/IL-4-treated group vs. SOD3-treated group).

The interactions of IL-4 with its receptor are known to activate JAK-STAT signaling pathways [23,24], and the engagement of anti-CD40 and LPS with their respective receptors are known to activate multiple downstream signals [4,5,25,26]. Thus, we examined the effect of SOD3 on various downstream signals. Here, we found that SOD3 suppressed IL-4-mediated activation of JAK1, JAK3, and STAT6 signals in B cells. Similarly, we also determined the effect of SOD3 on LPS or anti-CD40-mediated activation of p38, JNK, and NF-κB, and found that SOD3 significantly downregulates the activation of those molecules (Fig. 6A and B).

## 4 Discussion

Despite the important role of IgE in confronting helminthic infection, aberrant production of IgE was found to be associated with several allergic diseases [1,2]. The crosslinking of allergens to IgE antibodies on tissue mast cells or blood basophils results in the degranulation of these cells, and the release of inflammatory mediators, cytokines, and enzymes that mediate the clinical manifestation of various allergic conditions [27]. Previously, SOD3 has been shown to reduce the serum IgE levels in various allergic diseases [9,14,17]. However, the direct role of SOD3 in the production of IgE in B cells was not investigated. Here, we investigate the effect of SOD3 on IgE production and the effects on underlying signaling in B cells.

For the activation and differentiation of B cells into IgE secreting B cells, we used two different types of antigens. Lipopolysaccharides at high concentrations are capable of T-independent activation of B cells and the use of anti-CD40 ligand activates the B cells in T-dependent manner [28]. The use of IL-4 stimulates the class switching from IgM to IgE producing B cells [3,29]. In this study, we found that SOD3 significantly suppresses the release of IgE in B cells under both LPS/IL-4 and anti-CD40/IL-4 stimulating conditions. The higher production of IgE in B cells isolated from global SOD3 knock-out (*SOD3*^*−/−*^) mice compared to *SOD3*^*+/+*^ and the use of SOD3 limiting the release of IgE in both *SOD3*^*−/−*^ and *SOD3*^*+/+*^ mice emphasized the important role of SOD3 in regulating the production of IgE. Similarly, the use of SOD3 activity inhibitor DETCA tried to rescue the inhibitory effect of SOD3 on IgE production in B cells isolated from both *SOD3*^*−/−*^ and *SOD3*^*+/+*^ mice. However, DETCA could not completely reverse the inhibitory effect of SOD3. The possible reason is that SOD3 is also known to possess some effect in a non-enzymatic manner. Along with scavenging oxidative stress during inflammatory conditions, SOD3 also found to interact with receptors such as histamine 4 receptor in keratinocytes and IL-4 receptor in Th2 cells [30]. In addition, SOD3 found to inhibit the translocation of TLR into lipid rafts in DCs [31] and also inhibited the recruitment of NF-κB to the promoter site of target genes in macrophages [32] These non-enzymatic properties of SOD3 may be the possible reason that DETCA could not completely reverse the effect of SOD3 as DETCA is known only to inhibit the enzymatic properties of SOD3. Thus, we did not find any significant differences between SOD3 alone-treated groups and SOD3 with DETCA treated groups.

SOD3 has been shown to suppress the proliferation of Th17 cells [33]. Similarly, Th17 from *SOD3*^*−/−*^ mice showed more proliferation than *SOD3*^*+/+*^ mice [34]. In addition, cell cycle genes in MSCs under serum starvation and UVB-induced proliferation of melan-a cells were suppressed on SOD3 treatment [35,36]. These studies emphasized the importance of SOD3 in the regulation of proliferation. The cross-linking of anti-CD40 ligand and LPS with their respective receptors induces a proliferative response in B cells [28]. Upon antigenic stimulation, resting B cells promote from G0 and enter into G1 and S phase of the cell cycle, and undergo rapid proliferation [37]. The antigenic-induced cell cycle entries are tightly regulated by MAPK and NF-κB signaling pathways [38]. Our data showed that SOD3 can suppress the antigenic-mediated proliferation in B cells. However, these inhibitory effects of SOD3 on B cell proliferation may be mediated through controlled regulation of downstream signaling pathways such as MAPK and NF-κB required for cell cycle progression.

Upon activation by antigen, stimulated B cells undergo class switch recombination (CSR) process in which the immunoglobulin heavy μ constant region exons (Cμ) are deleted and replaced by one of several sets of CH exons (e.g., Cγ, Cα, and Cε) to alter the isotype of the antibodies, resulting in the production of specific antibodies [39,40]. To undergo isotype switching for IgE production, B cells require two distinct signals [5]. The first signal is provided through the binding of IL-4 to its receptor on the B cell which induces Cε germline transcripts (GLTs) [29]. The second signal is provided through ligation of CD40 on the B cell surface by CD40 ligand of T cells and triggers the deletional switch recombination to IgE [41]. Stimulation of B cells with anti-CD40 and IL-4 results in the activation of NF-κB and STAT6 transcription factors respectively, and functions to induce the production of Cε GLTs [18,42]. The Iε promoter contains the binding sites for STAT6 and NF-κB, and the binding of these transcription factors to the promoter results in the induction of Cε germline transcripts [43]. The activation-induced cytidine deaminase (AID) enzyme is required for CSR [44]. Ligations of CD40-induced NF-κB synergize with IL-4-induced STAT6 stimulates the expression of AID gene and proteins [45]. Our data showed that SOD3 significantly downregulates the expression levels of genes involved in CSR and AID. However, this inhibitory effect of SOD3 on IgE switch recombination may be mediated through reduced activation of downstream NF-κB and STAT6 transcription factors.

Upon activation, B cell is not much known to secrete various cytokines as T cells. The main cytokines produced by B cells are the chemokines such as CCL17 and CCL22 ^20–22^. These chemokines function to recruit Th2 cells and in turn, Th2 cells produce cytokines required for B cell differentiation and antibody production [46]. Here, we found that treatment with SOD3 suppressed the secretion of chemokines CCL17 and CCL22 in B cells. This result illustrates the possible mechanism of reduced expression of IgE levels *in vivo*, where cytokines produced by Th2 cells and B cells work synergistically for the secretion of IgE.

Activated B cells are known to differentiate into antibody-secreting cells, leading to adaptive immune response through the secretion of antibodies. Following ligand interaction with either Toll-like receptor (TLR) or with B cell receptor, B cells rapidly undergo proliferation and differentiate into antibody-secreting cells [28,38]. Our data showed that treatment with SOD3 inhibited both LPS/IL-4 and anti-CD40/IL-4-driven differentiation of IgE secreting cells analyzed with ELISpot assays. Similarly, engagement of B cell antigen receptors results into the upregulation of ROS, and the production of ROS is important for the differentiation and activation of downstream signaling events [47,48]. Here, we found that SOD3 suppressed the ROS levels in B cells. Thus, the inhibition of IgE secreting cells may be mediated through controlled regulation of LPS/IL-4 or anti-CD40/IL-4-induced activation of downstream signaling events.

**Fig. 7:**
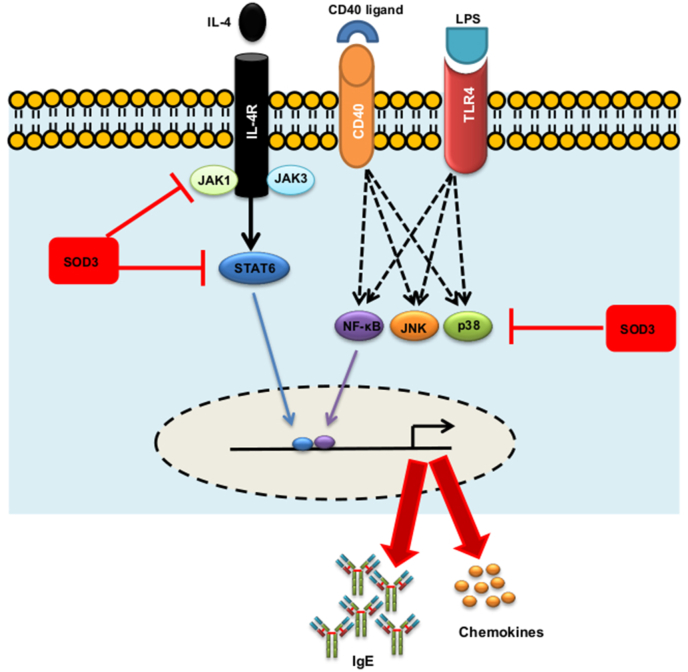
**A proposed mechanism of SOD3 on IgE production in B cells.** Ligations of LPS or anti CD40 along with IL-4 on B cells trigger the activation of JAK-STAT, p38, JNK, and NFκB signaling events which result in the secretion of IgE and chemokines. SOD3 suppressed the activation of these cellular signals and thus controls the release of IgE and chemokines in B cells.

Ligation of LPS and CD40 ligand with TLR4 and CD40 receptors respectively on B cells are known to activate downstream signaling pathways such as MAPK and NF-κB and subsequently leads to the activation and proliferation of B cells [4,49]. Similarly, engagement of IL-4 with IL-4 receptor on B cells recruit Janus kinases, JAK1 and JAK3, which causes the activation and nuclear translocation of STAT6 transcription factor [24,50]. Binding of NF-κB and STAT6 transcription factors to their promoter regulate AID induction and IgE class switching [18,42]. Several studies showed that SOD3 can significantly modulate the activation of cellular signaling events through both enzymatic and non-enzymatic manner [[8], [9], [10],^17^,^51^]. A study by Kwon et al. showed that SOD3 inhibits the translocation of TLR4 into lipid rafts in dendritic cells (DCs) and also inhibited the recruitment of NF-κB to the promoter site of target genes in macrophages [8]. Interestingly, we have shown that SOD3 can interact with IL-4 receptor in Th2 cells [11]. Thus, suppressed activation of IL-4-mediated JAK-STAT signaling pathways may be mediated through the interaction of SOD3 with IL-4 receptor. Taken together, we believe that SOD3 can modulate the activation of these signaling events and thereby controls the production of IgE and chemokines secretion in B cells (Fig. 7).

Plethora of studies suggests that SOD3 can be used as novel treatment strategy for various diseases such as cancer, diabetes, rheumatoid arthritis, chronic inflammation, aging and neurodegenerative diseases [52]. Previous studies showed that treatment with SOD3 showed reduced serum level of OVA-induced IgE in experimental allergic conjunctivitis and ovalbumin-induced allergic asthma murine models [32,53]. Similarly, the treatment with SOD3 also found to suppress the serum IgE levels in house dust mite-induced atopic dermatitis-like skin inflammation [14]. Our data showed that, SOD3 can inhibit the production of IgE in B cells. Thus, previous studies along with our current findings emphasized the importance of SOD3 as an alternative therapy to restrict IgE-mediated allergic diseases.

## 5 Conclusion

Along with antioxidant and anti-inflammatory functions, SOD3 also has been shown to possess immunomodulatory functions in various immune cells. SOD3 was found to suppress DCs maturation through reduced expression of receptor proteins [8]. Similarly, SOD3 was also found to restrict T helper cell activation and differentiation [9,34]. In addition, SOD3 was found to inhibit the cathelicidin- and IgE-mediated degranulation of mast cells [14,51]. Previous studies showed that SOD3 significantly downregulated the serum IgE levels in various disease mouse models [[12], [13], [14]]. These studies along with our current study emphasized the importance of SOD3 in regulating immunomodulatory functions. However, extensive studies must be carried out for the effective use of SOD3 as an alternative treatment for IgE-mediated allergic conditions.

## Author contributions

GA contributed to the design, conceptualization, formal analysis, investigation, methodology, visualization, and writing; SKS, MJL, CWB, YHK and HYK contributed to the analysis, and writing; TYK contributed to conceptualization, supervision, and validation. All authors reviewed the manuscript.

## Data availability statement

All data generated or analyzed during this study are included in this article (and its supplementary information files).

## Declaration of competing interest

The authors declare that they have no known competing financial interests or personal relationships that could have appeared to influence the work reported in this paper
