# Supplementary material for: Inhibitory effects of superoxide dismutase 3 on IgE production in B cells
Source: Biochem Biophys Rep. 2022 Feb 3;29:101226. doi: 10.1016/j.bbrep.2022.101226 (PMC8822298; doi:10.1016/j.bbrep.2022.101226)
Supplement: Multimedia component 1 [file mmc1.pdf]

Inhibitory effects of Superoxide dismutase 3 inhibits on IgE production in B cells

Gaurav Agrahari[1], Shyam Kishor Sah[2], Chul Hwan Bang[1], Yeong Ho Kim[1] Tae-Yoon Kim[1*]

[1]Laboratory of Dermato-Immunology, College of Medicine, The Catholic University of Korea, 06591, Seoul, Republic of Korea

[2]Department of Reconstructive Sciences, Center for Regenerative Medicine and Skeletal Development, UConn Health, USA

This pdf incudes:

Supplementary figure: S1to S4

Supplementary figure legends

Figure S1

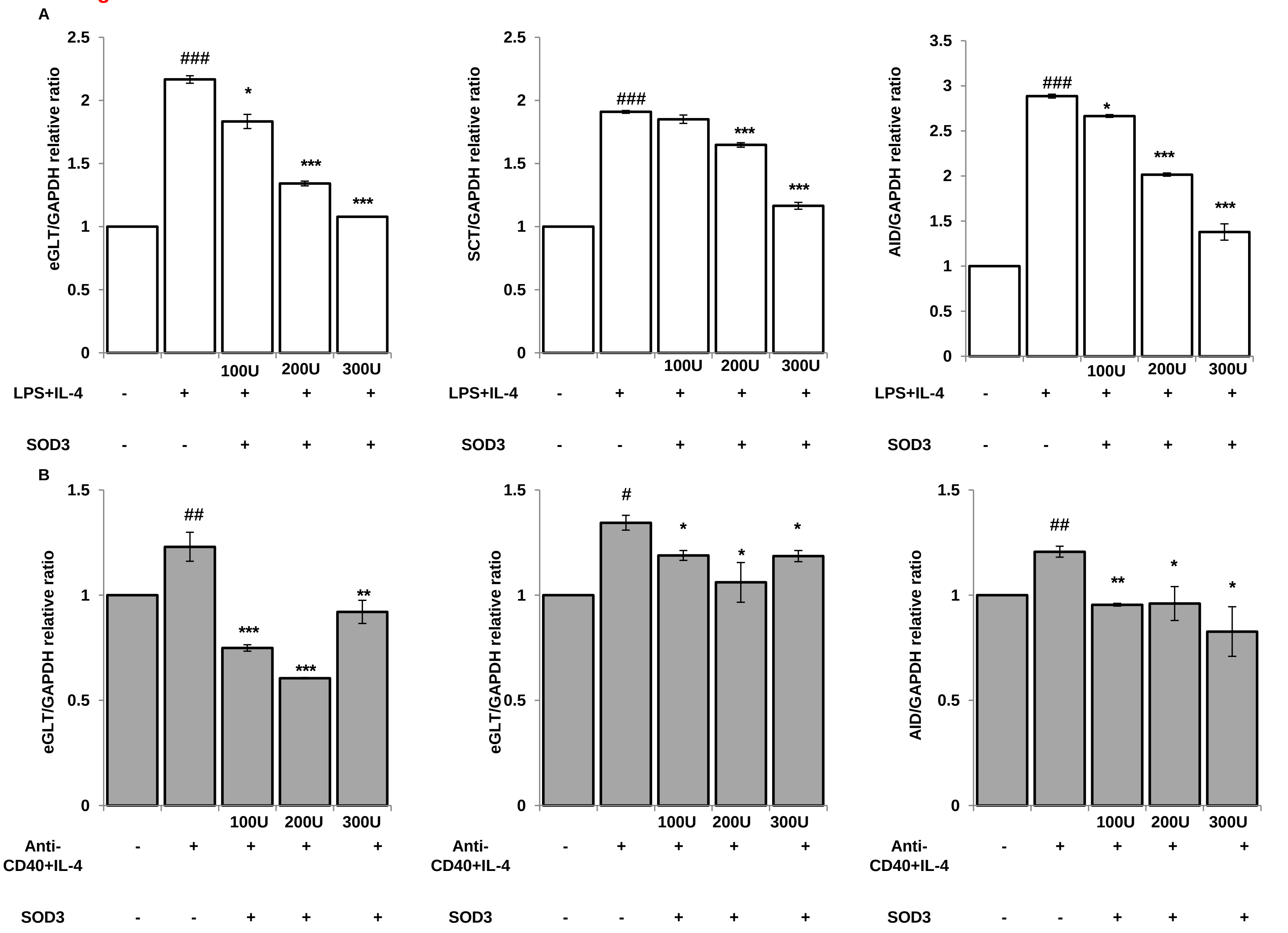
A
eGLT/GAPDH relative ratio
SCT/GAPDH relative ratio
AID/GAPDH relative ratio
###
*
***
100U
200U
300U
LPS+IL-4
SOD3
B
eGLT/GAPDH relative ratio
AID/GAPDH relative ratio
##
#
**
Anti-CD40+IL-4
SOD3

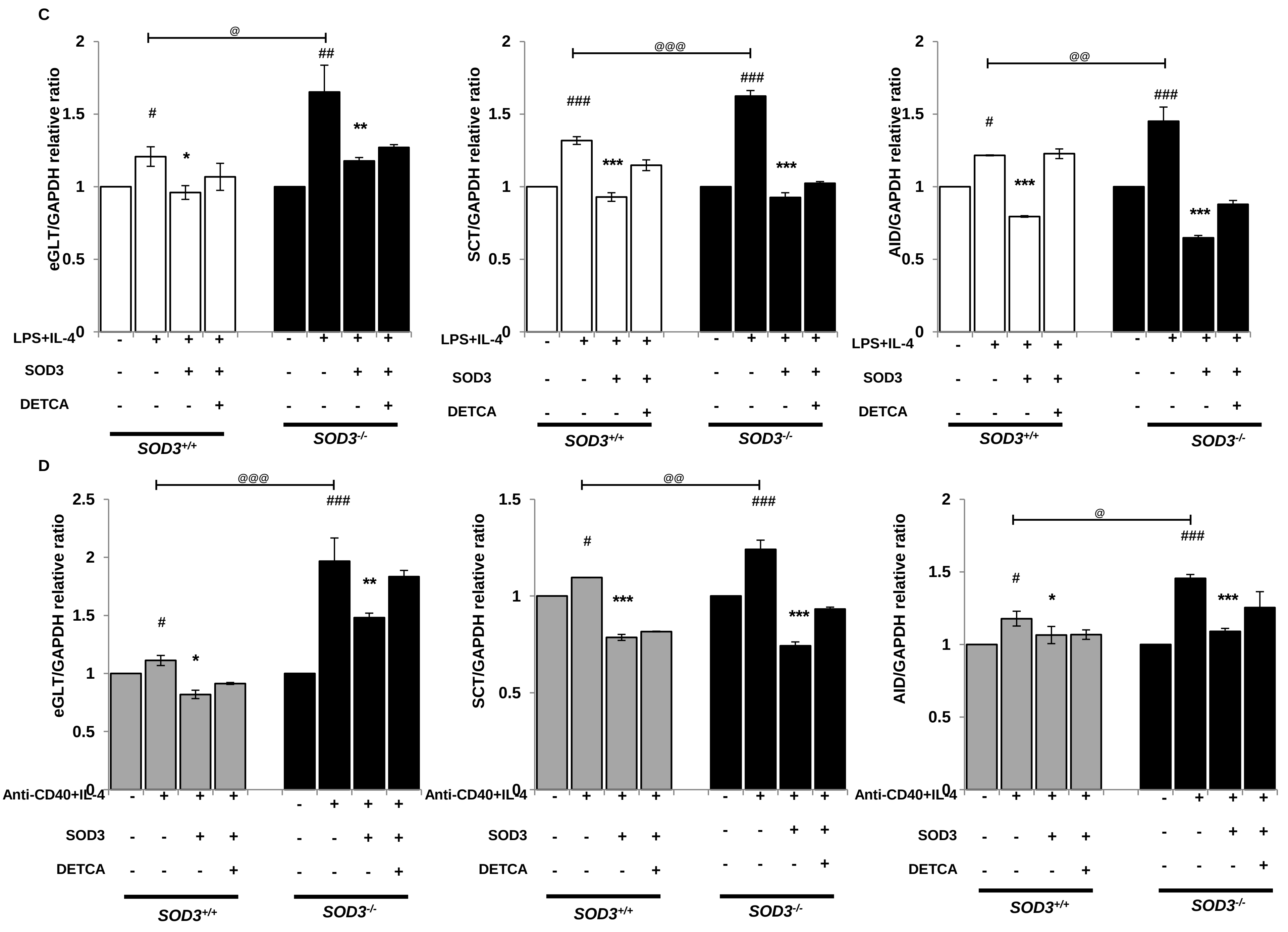
C
eGLT/GAPDH relative ratio
SCT/GAPDH relative ratio
AID/GAPDH relative ratio
LPS+IL-4
SOD3
DETCA
$SOD3^{+/+}$
$SOD3^{-/-}$
D
Anti-CD40+IL-4
SOD3
DETCA
$SOD3^{+/+}$
$SOD3^{-/-}$

Figure S2

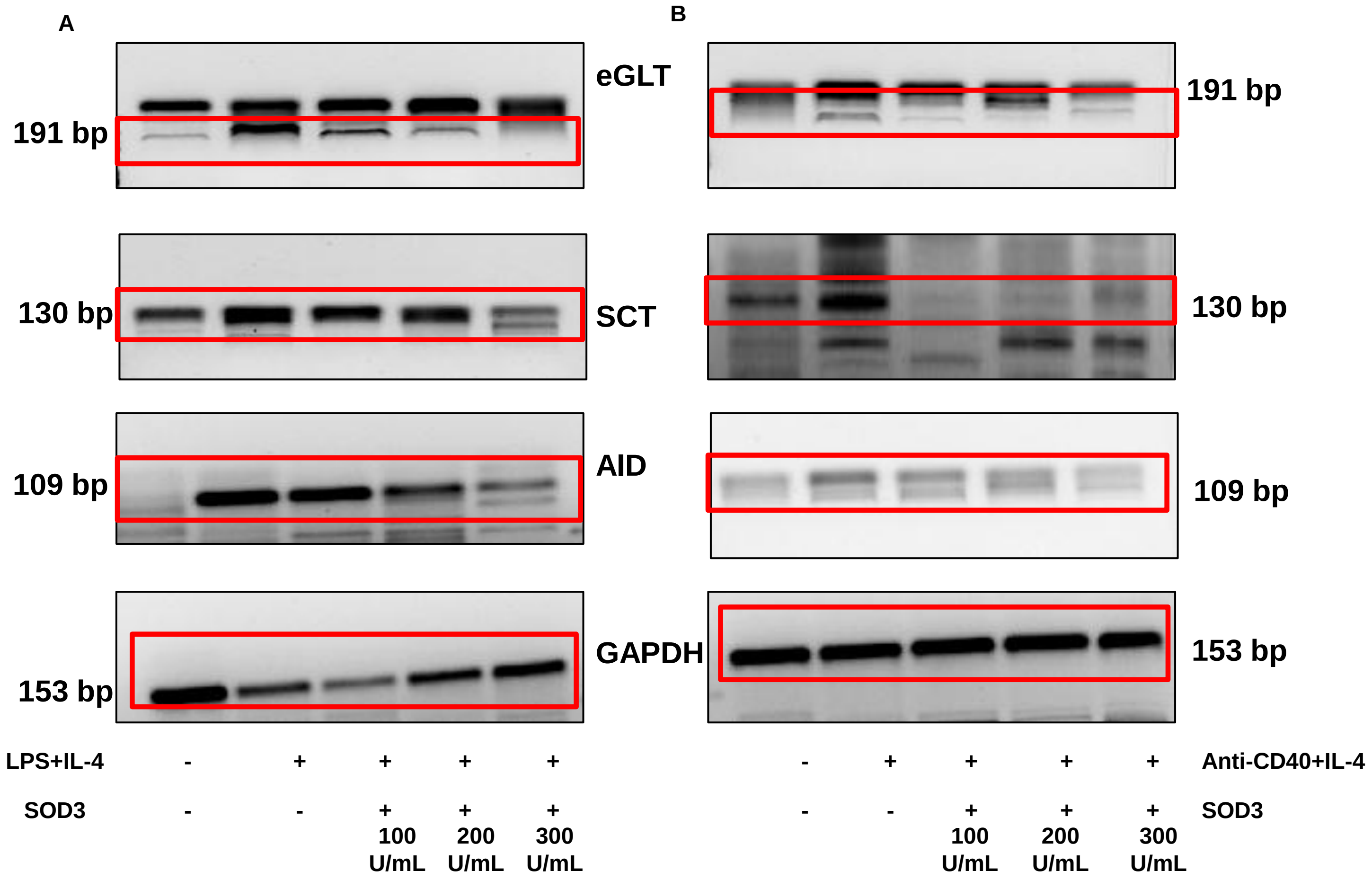
A
B
eGLT
SCT
AID
GAPDH
191 bp
130 bp
109 bp
153 bp
191 bp
130 bp
109 bp
153 bp
LPS+IL-4
SOD3
- + + + +
- - + + +
100 U/mL
200 U/mL
300 U/mL
Anti-CD40+IL-4
SOD3
- + + + +
- - + + +
100 U/mL
200 U/mL
300 U/mL

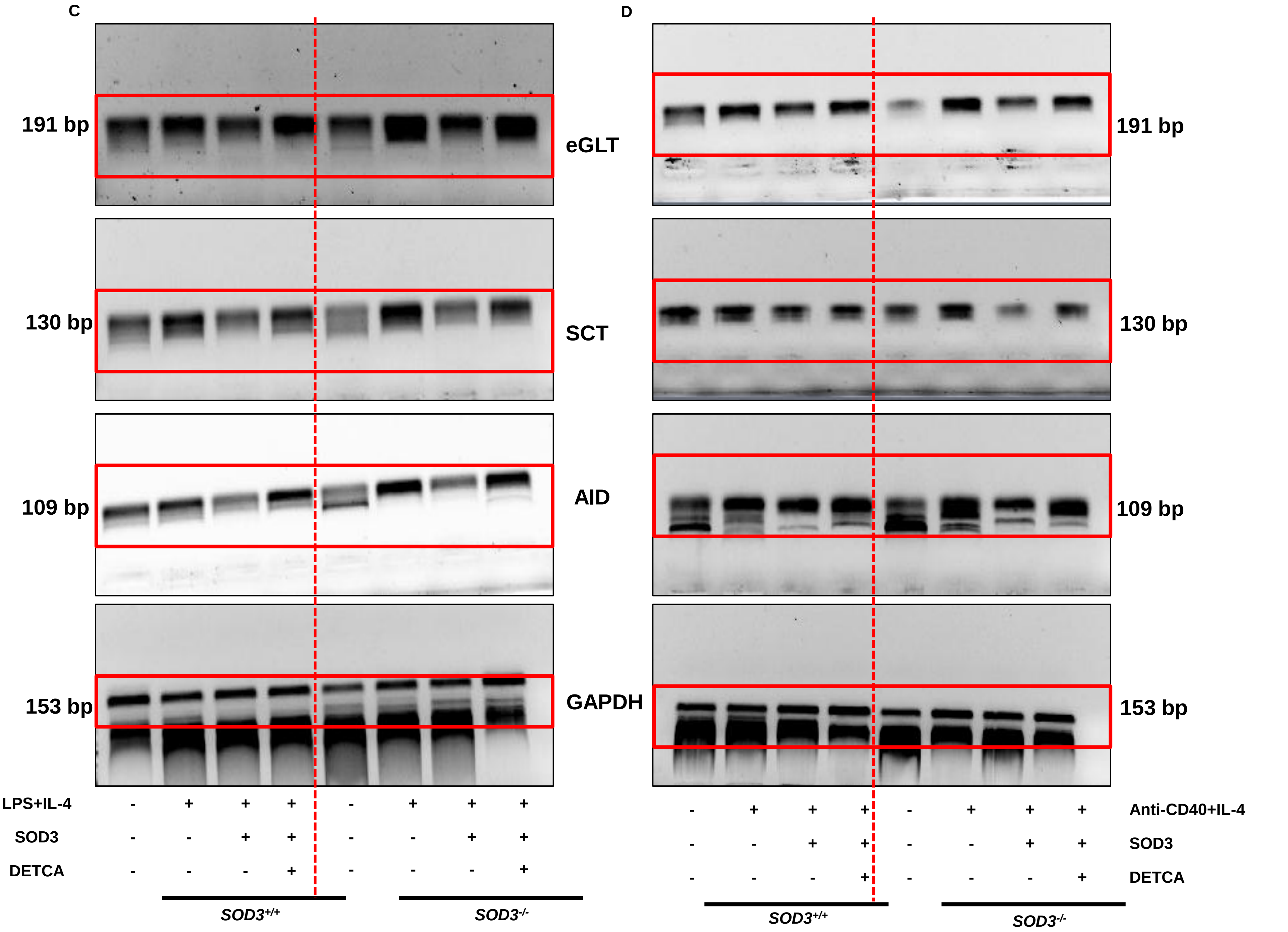
C
191 bp
eGLT
130 bp
SCT
109 bp
AID
153 bp
GAPDH
LPS+IL-4 - + + + - + + +
SOD3 - - + + - - + +
DETCA - - - + - - - +
SOD3+/+
SOD3-/-
D
191 bp
130 bp
109 bp
153 bp
- + + + - + + + Anti-CD40+IL-4
- - + + - - + + SOD3
- - - + - - - + DETCA
SOD3+/+
SOD3-/-

Figure S3

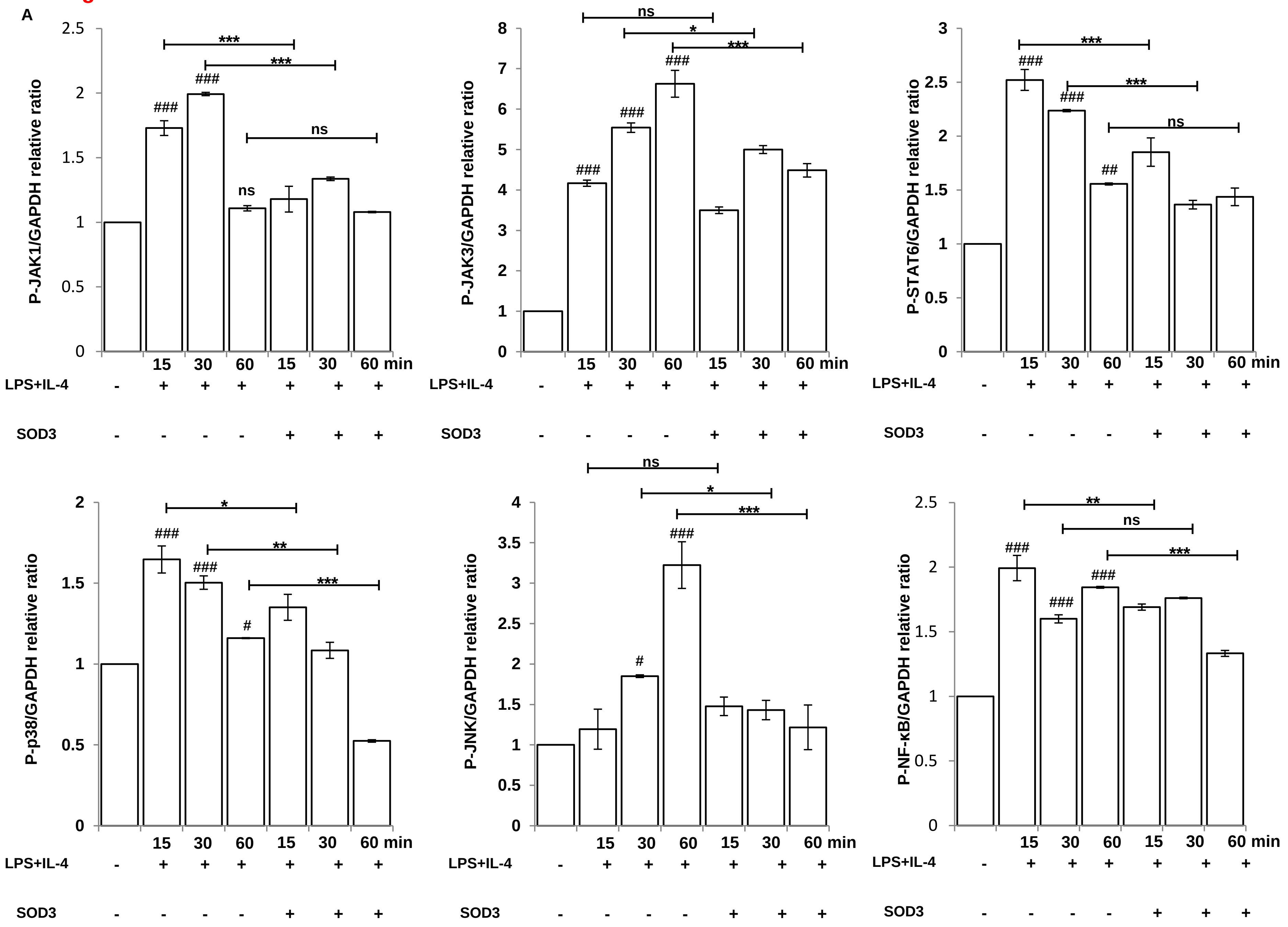
A
P-JAK1/GAPDH relative ratio
P-JAK3/GAPDH relative ratio
P-STAT6/GAPDH relative ratio
P-p38/GAPDH relative ratio
P-JNK/GAPDH relative ratio
P-NF-κB/GAPDH relative ratio
15 30 60 15 30 60 min
LPS+IL-4 - + + + + + +
SOD3 - - - - + + +
###
##
#
***
**
*
ns

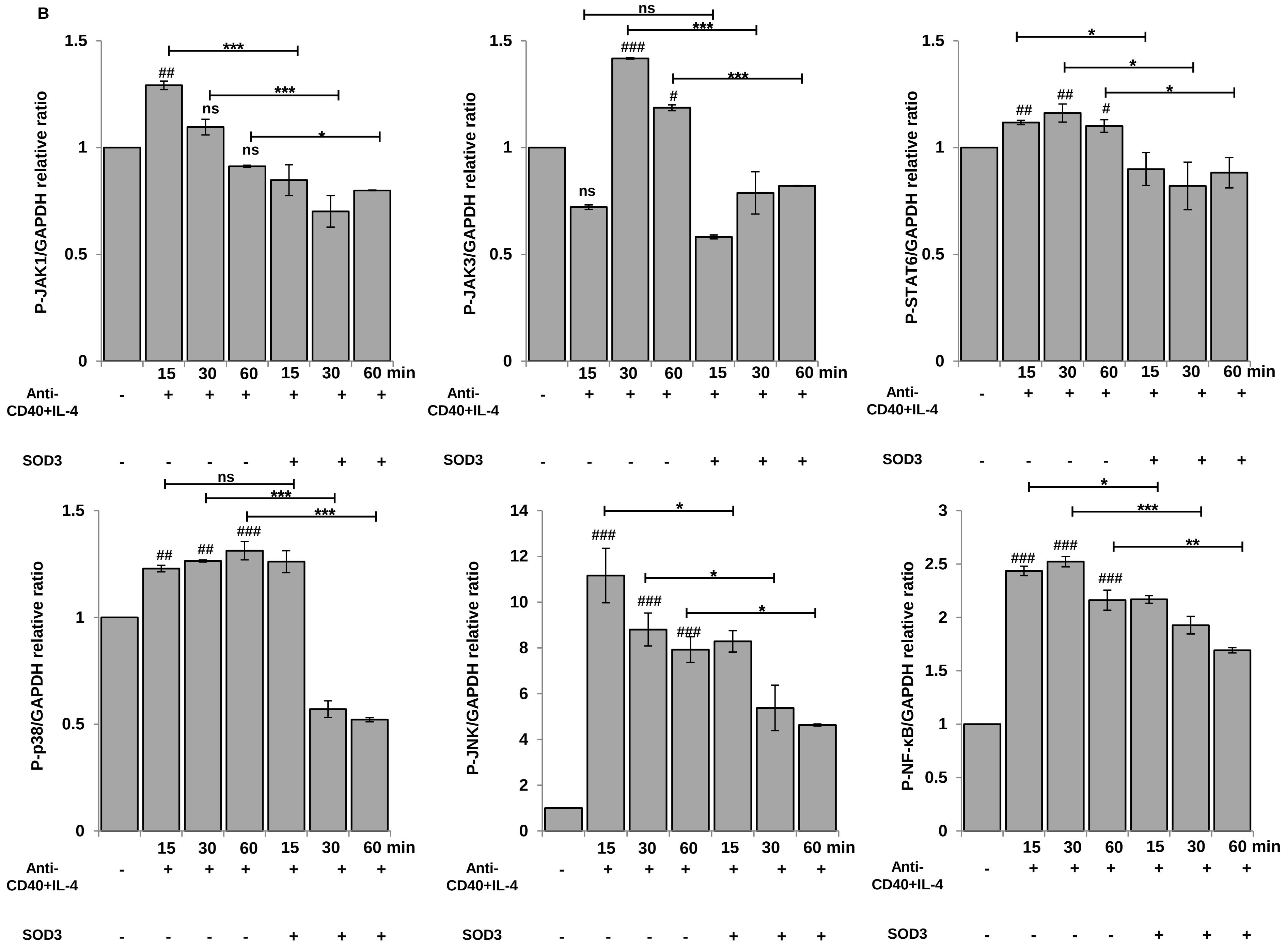

B
P-JAK1/GAPDH relative ratio
P-JAK3/GAPDH relative ratio
P-STAT6/GAPDH relative ratio
P-p38/GAPDH relative ratio
P-JNK/GAPDH relative ratio
P-NF-κB/GAPDH relative ratio
15 30 60 15 30 60 min
Anti-CD40+IL-4 - + + + + + +
SOD3 - - - - + + +
ns
***
*
##
#
###
**

Figure S4

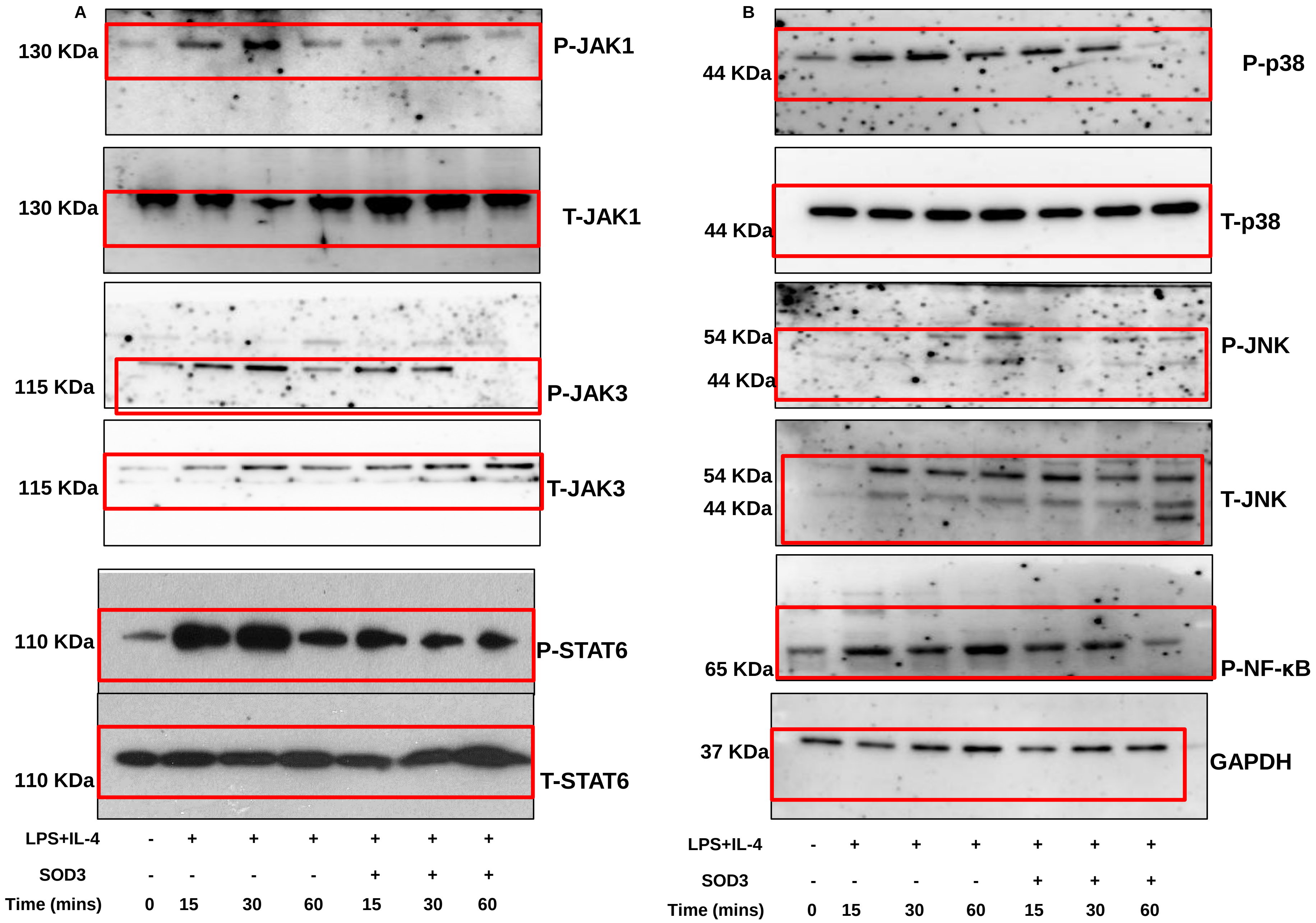

A
130 KDa
P-JAK1
130 KDa
T-JAK1
115 KDa
P-JAK3
115 KDa
T-JAK3
110 KDa
P-STAT6
110 KDa
T-STAT6
LPS+IL-4 - + + + + + +
SOD3 - - - - + + +
Time (mins) 0 15 30 60 15 30 60
B
44 KDa
P-p38
44 KDa
T-p38
54 KDa
44 KDa
P-JNK
54 KDa
44 KDa
T-JNK
65 KDa
P-NF-κB
37 KDa
GAPDH
LPS+IL-4 - + + + + + +
SOD3 - - - - + + +
Time (mins) 0 15 30 60 15 30 60

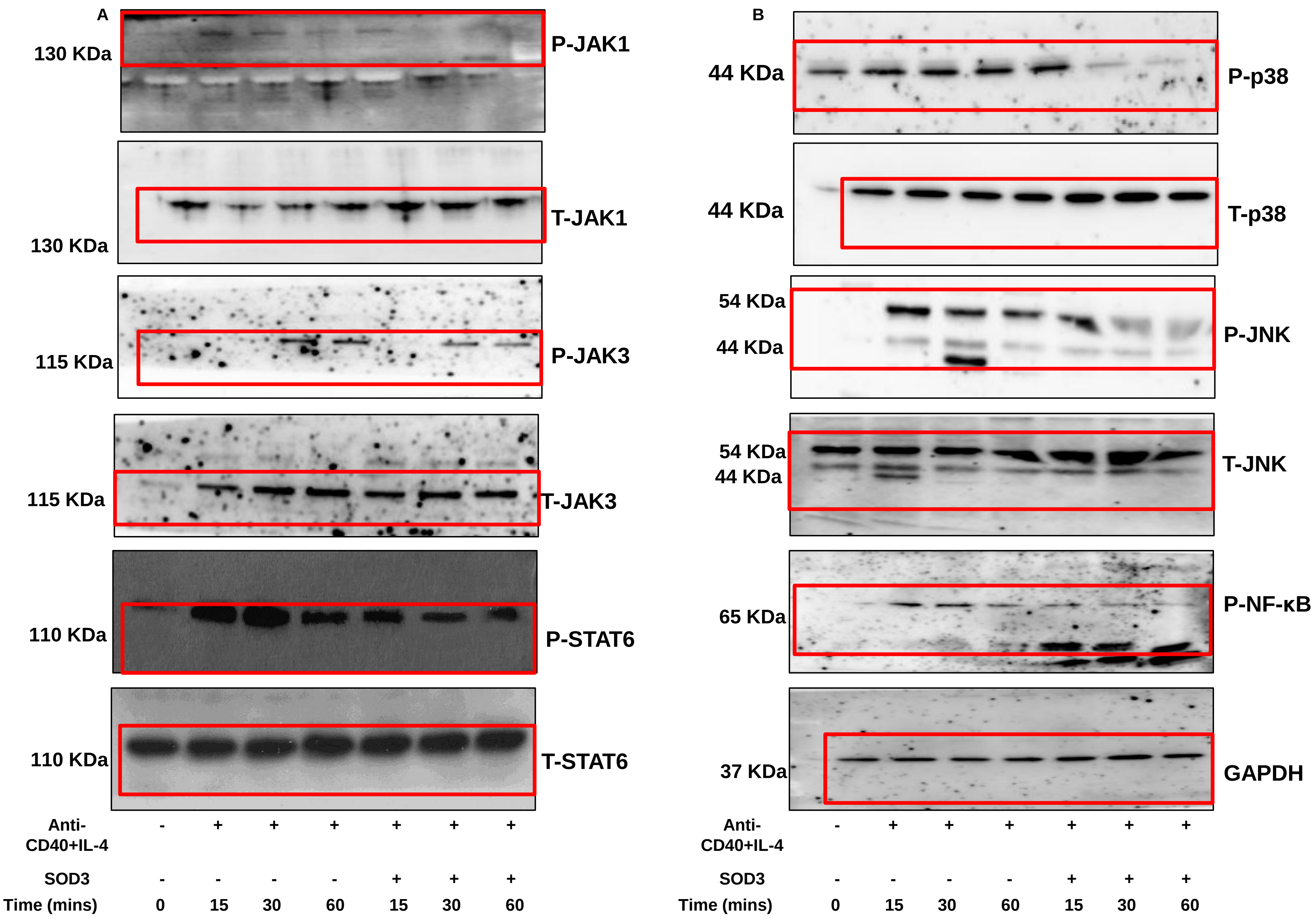
A
130 KDa
P-JAK1
T-JAK1
130 KDa
115 KDa
P-JAK3
115 KDa
T-JAK3
110 KDa
P-STAT6
110 KDa
T-STAT6
Anti-CD40+IL-4 - + + + + + +
SOD3 - - - - + + +
Time (mins) 0 15 30 60 15 30 60
B
44 KDa
P-p38
44 KDa
T-p38
54 KDa
44 KDa
P-JNK
54 KDa
44 KDa
T-JNK
65 KDa
P-NF-κB
37 KDa
GAPDH
Anti-CD40+IL-4 - + + + + + +
SOD3 - - - - + + +
Time (mins) 0 15 30 60 15 30 60

Supplementary figure legends

Figure S1: Band intensities of gel electrophoresis blot. ImageJ (version 1.46r) software was used to determine the band intensities of gel blot data from (A) Figure 3A, (B) Figure 3B, (C) Figure 3C, (D) Figure 3D. All experiments were performed in triplicate. Data are expressed as mean ± standard deviation. $^{\#}p<0.05$, $^{\#\#}p<0.01$, $^{\#\#\#}p<0.001$ (Control group vs. LPS/IL-4 or anti-CD40/IL-4-treated group); $^{*}p<0.05$, $^{**}p<0.01$, $^{***}p<0.001$ (LPS/IL-4 or anti-CD40/IL-4-treated group vs. SOD3-treated group); $^{@}p<0.05$, $^{@@}p<0.01$, $^{@@@}p<0.001$ ($SOD3^{+/+}$vs $SOD3^{-/-}$).

Figure S2: Full length blots of gel electrophoresis data of (A) Figure 3A, (B) Figure 3B, (C) Figure 3C, (D) Figure 3D.

Figure S3: protein band intensities of western blot. ImageJ (version 1.46r) software was used to determine the band intensities of western blot data from (A) Figure 6A, (B) Figure 6B. All experiments were performed in triplicate. Data are expressed as mean ± standard deviation. .$^{\#}p<0.05$, $^{\#\#}p<0.01$, $^{\#\#\#}p<0.001$ (Control group vs. LPS/IL-4 or anti-CD40/IL-4-treated group); $^{*}p<0.05$, $^{**}p<0.01$, $^{***}p<0.001$ (LPS/IL-4 or anti-CD40/IL-4-treated group vs. SOD3-treated group.

Figure S4: Full length blots of western blot data of (A) Figure 6A, (B) Figure 6B.
