# Supplementary material for: Inhibitory effects of superoxide dismutase 3 on IgE production in B cells
Source: Biochem Biophys Rep. 2022 Feb 3;29:101226. doi: 10.1016/j.bbrep.2022.101226 (PMC8822298; doi:10.1016/j.bbrep.2022.101226)
Supplement: Multimedia component 2 [file mmc2.docx]

| Primer | Forward | Reversse |
| --- | --- | --- |
| **eGLT** | **GAGATTCACAACGCCTGG** | **CTTTACAGGGCTTCAAGGG** |
| **SCT** | **CTGGCCAGCCACTCACTTAT** | **AGGTGAAGGAAATGGTGCTC** |
| **AID** | **AGTCACGCTGGAGACCGATA** | **GCAGAGGTAGGTCTCATGCC** |
| **GAPDH** | **CATCACTGCCACCCAGAAGACTG** | **ATGCCAGTGAGCTTCCCGTTCAG** |

**Table S1:** Primer sequences for RT-PCR of mRNA.
